# Energizing compassion: using music and community focus to stimulate compassion drive and sense of connectedness

**DOI:** 10.3389/fpsyg.2023.1150592

**Published:** 2023-10-04

**Authors:** Paul Gilbert, Jaskaran Kaur Basran, Ptarmigan Plowright, Hannah Gilbert

**Affiliations:** ^1^Centre for Compassion Research and Training, College of Health, Psychology and Social Care, University of Derby, Derby, United Kingdom; ^2^The Compassionate Mind Foundation, Derby, United Kingdom; ^3^University of Roehampton, London, United Kingdom

**Keywords:** compassion, meditation, energizing, connectedness, music, tonglen

## Abstract

**Objectives:**

The last 20 years have seen considerable research on the nature and biopsychosocial impacts of compassion training on self and others. This training is usually focused on calming and slowing the mind and body and on individual imagery practices and mantras. This study explored the effects of three variations: 1. The impact of using energizing music to generate activation and “drive” for compassion; 2. To focus on imagining “breathing in and breathing out a white light or mist of compassion” to bring compassion to the world; and 3. While listening to energizing music, participants were guided to imagining connecting to the compassion (Sangha) community, imagining oneself as linking with others as part of communities seeking to help the world.

**Methods:**

From approximately 1,600 members of the Compassionate Mind discussion list, participants were invited to take part in a new energizing focused self-practice study. The study involved listening to recorded guidance on the evolutionary model of compassion and the need to address the potentially harmful side of our nature. This was followed by a 4 1/2-min tonglen-informed guided practice of breathing in and breathing out compassion accompanied by energizing music. Forty-three participants completed several self-report scales measuring compassion orientation, wellbeing, social safeness, and positive affect before and following 2 weeks of practice. Participant experiences were recorded from 6 open explorative questions.

**Results:**

Self-report measures taken before and following 2 weeks of practice revealed significant increases in self-compassion, compassion to others, openness to compassion from others, activated positive affect, safe positive affect, social safeness, and wellbeing, with the largest effect size relating to compassion for the self (*d* = −0.76). In addition, qualitative data revealed that the participants had experienced the practice as energizing, inspiring, and felt socially connected and that it had significant impacts on other aspects of their lives. Some participants noted that engaging with suffering also stimulated sadness.

**Conclusion:**

This study found that pairing energizing music with breathing practices and specific compassion visualizations, focusing on the desire to bring compassion to the world and be part of a compassionate community, was well-accepted and had a range of significant positive impacts. This study indicates the potential value of exploring energizing in comparison to the more standard soothing and settling practices as ways of stimulating the biopsychosocial processes of compassion.

## Introduction

The biopsychosocial benefits of cultivating compassion have been promoted for thousands of years (Dalai Lama, 1995; Lampert, 2005; Ricard, 2015). More recently, the nature and beneficial impact of compassion has come under scientific exploration (for reviews see Gilbert, 2017; Kirby, 2017; Seppälä et al., 2017; Roca et al., 2021). Although there remain controversies and variations in how compassion is defined and measured (Mascaro et al., 2020), rooting compassion in its evolved algorithm (Gilbert, 2009, 2014, 2017, 2020a,b; Gilbert and Choden, 2013) offers a fairly standard motive-based definition that compassion constitutes *a sensitivity to suffering in self and others with a commitment to try to alleviate and prevent it* (Dalai Lama, 1995; Goetz et al., 2010; Gilbert, 2017; Mascaro et al., 2020). The advantage of seeing compassion as a stimulus–response algorithm (i.e., “*if* A *then* do B”) is that it enables the identification of two different elements. These are first to explore the processes that facilitate people's detection and preparedness to move toward and engage with suffering, and second, the processes that influence people's efforts to work out what to do and actually do them (Gilbert, 2009; Poulin, 2017; Di Bello et al., 2020). This means that the first movements to compassion can be stressful because we are moving toward pain or threat (Gilbert, 2009; Di Bello et al., 2020). Studies have shown that when only distress is focused on (e.g., through images or stories), compassion can be stressful (Gilbert et al., 2017; Condon and Makransky, 2020; Di Bello et al., 2020). Condon and Makransky (2020) have drawn attention to this issue and developed what they call *sustainable compassion training*. Like Compassion Focused Therapy (CFT), they suggested that training in compassion needs to teach abilities to be sensitive and have the courage and wisdom for skilful engagement but also ways to be helpful. Hence, the second element of the compassion algorithm is the action and response function. When guiding people in compassion, it is important how and what people learn about compassion (Mascaro et al., 2022).

Planning and taking action requires a different set of skills and a different type of empathy to work out what will be helpful and to act on it compared to being sensitive, moving into and empathizing with suffering. Planning and taking action are also related to different physiological processes (Di Bello et al., 2020). Poulin (2017) notes that people can be motivated and knowledgeable of what to do but still not take compassionate action. The skills of compassion can also differ with context. For example, a skilled firefighter, social advocate, or therapist counseling a dying client require different types of empathic skill, tolerance, and other compassion competencies, but are united in *the motive* to try to address suffering in their context. This means individuals can train in specific competencies for specific contexts. Individuals who can behave compassionately in one context, for example, risking their lives to save others, may not be that empathic or compassionate in another context, such as having empathic sensitivity to mental distress. We should also note that our use of competencies to be sensitive to suffering and its causes are related *to motives*. For example, the motives for vengeance, cruelty, or sadistic enjoyment can also involve sensitivity to suffering, but how to cause it rather than relieve it.

The fact that the algorithm of compassion has two very distinct processes complicates how we investigate its social and psychophysiological processes, particularly when we are exploring people's reactions to distress or their planned actions which are context-dependent. Di Bello et al. (2020) studied subjective and physiological responses to two videos. Video 1 invited participants to look at individuals in distress and explored empathic sensitivity. Video 2 invited participants to look at people engaging in helpful actions. Following the first video, participants experienced an increase in sadness and a decrease in positive affect, as well as a decrease in vagally-mediated heart rate variability (vmHRV). This shows that the first aspect of compassion (engagement with and sensitivity to suffering) involves empathic resonance and a decrease in one's own positive emotions. After participants watched the second video, which tapped into the “action” component of compassion, a decrease in sadness and an increase in vmHRV was found. The results, therefore, indicate how the two processes of compassion are linked to different psychophysiologies.

Loving-kindness meditations mitigate against the problems of only being sensitive to distress because they focus on distress but then quickly shift the participant to the response component of wishing for a positive outcome for the person. For example, Weng et al. (2018) offered instructions:

*For each person, they imagined a time when the person had suffered, brought non-judgmental and balanced attention to reactions to suffering, and then practiced wishing the person relief from suffering. They repeated compassion-generating phrases such as, “May you be free from suffering. May you have joy and happiness.” They were also instructed to pay attention to bodily sensations (particularly around the heart) and to envision a golden light extending from their heart to the heart of the other person* (p. 4).

The rapid movement from awareness of suffering to positive responses with the wish to be free of suffering stimulates different physiological systems (Petrocchi et al., 2022).

Many of these forms of meditative practices also focus on mindfulness and processes of *slowing, soothing, and grounding* in the body (Weng et al., 2013, 2018). These guided practices seek to stimulate the vagus nerve and other physiological infrastructures that support compassion (Keltner et al., 2014; Porges, 2017; Kirschner et al., 2019).

### Energizing and music

Soothing effects may be a result of the way that training is conducted because compassion can also increase arousal (Di Bello et al., 2020). Indeed, compassionate action often requires invigorating behavior, for example, in saving others or struggling for social justice and taking heroic action (Zimbardo, 2019). We wanted to explore the impact of a different type of compassion practice that deliberately seeks to activate rather than calm. There are spiritual practices, such as the use of Sufi whirling and other forms of dance, that seek to create an experience of self-transcendence, and stimulate compassion with activation and arousal (Winton-Henry, 2009). Linked to a more “invigorated” approach to compassion, there is increasing evidence that certain kinds of energized movements, such as yoga and dance, can create a sense of interconnectedness that supports compassion motivation. They can invigorate feelings of encouragement, enthusiasm, and joy for wanting to spread compassion and take action (Gard et al., 2012; Karkou et al., 2019; Yilmazer et al., 2020).

We did not use dance in our study but we did use music that can energize and give people the desire to want to move or dance. We accompanied visualizations with energizing music taken from Thomas Bergersen called the Final Frontier, available on the internet (and used in this study with permission). There is good evidence that music can have a variety of impacts on emotional states as exemplified by how it is used in film scenes. There is also good evidence that music can have major therapeutic benefits (De Witte et al., 2020). For many years, one of the authors (PG) has introduced these practices to colleagues and participants during retreats and in training. It was based on their feedback and experience that the current practice was developed for research.

### Tonglen

One of the authors (PG) was introduced to several different practices of tonglen by a Buddhist monk called Choden and his colleagues during training in Samye Ling (Gilbert and Choden, 2013). It is seen mainly as a Tibetan Buddhist practice believed to be around 1,000 years old. It evolved to help promote compassion and the courage to engage with suffering and reduce ego-focusing. It invites a more visceral approach to take in the suffering of others and breathe out compassion, allied with a strong wish for suffering to be relieved, and forms part of Bodhicitta practice. A simple overview is given by Chödrön (2023), a more detailed description of the process by Berzin (2005), and some research studies by Mah et al. (2020). As noted, the standard method is to imagine breathing in suffering (sometimes in the form of dark smoke), imagine it being transformed in one's heart, and then breathing out a white light of compassion, with a focus on one's heartfelt wish for that to be healing. It heightens the issue of taking on the pain of others and transforming it. It also stimulates a sense of responsibility to address the suffering around us.

In this study, we changed the focus because imagining taking on or taking in the suffering of others is an advanced practice and we were more interested in focusing on the energizing process of bringing compassion into the world. So, instead of breathing in suffering, we invited participants to imagine breathing in a white light or mist of compassion, which fills one's body and invigorates compassion (in a more advanced practice, participants can imagine breathing in a bright white light that has emanated from an imagined Buddha sitting at the center of the universe who is emanating compassion and energy) (Rinpoche and Mullen, 2005). Then, participants were invited to imagine breathing out compassion in the form of white light or mist to address the suffering in the world. So, basically, participants imagined breathing in and breathing out compassionate light.

The focus of compassion is to address suffering and the causes of suffering. One of the causes of suffering is, of course, ourselves. One of the reasons we can be so harmful is because we are all evolved beings that did not choose to be here and have an evolved and socially shaped brain that can be tricky and harmful according to what gets activated. Looking back over the last few thousand years, it is clear that humans have a terrible dark side, with their history of wars, holocausts, torture, slavery, and everyday callousness (Gilbert, 2019b). While loving-kindness tends to focus on the wish for others to be free of suffering and happiness, another focus can be to bring the power of compassion to the dark side in symbolic processes or visualizations. One of the authors (PG) adapted the practice such that when we breathe out compassionate light, we imagine breathing out light to address the darkness, to light up the darkness as a way of focusing on addressing the dark side of humanity. Hence, participants ground themselves, using a standard soothing rhythm breathing practice, then imagine breathing in compassionate light and breathing out compassionate light. What they breathe out represents the light that brings enlightenment and compassion to the world because we are all born with tricky brains and can do harmful things.

### Part of a community

One of the most important evolutionary adaptations for humans is our extraordinary capacity to do things together and to want to feel part of a community and have a sense of belonging (Baumeister and Leary, 1995; Mikulincer and Shaver, 2014; Camilleri et al., 2023). In many Buddhist traditions, learning and practicing meditation began in communities and monasteries (the Sangha) and only later, when individuals practiced, would they spend more time meditating alone. We believe that visualizing oneself as part of a community that shares the collaborative wish to bring compassion into the world, also stimulates courage for compassion via a sense of belonging and joint action. Hence, the second part of this visualization invited participants to consider that they were amongst others doing the same practice. Additionally, towards the end of the practice, participants were asked to imagine that the compassionate light they were breathing out would coalesce with that of others to become an expanse of compassionate light spreading into the darkness.

Compassion has been studied in different ways including through physiology, behavior, and self-report measures (see Seppälä et al., 2017). As this was an internet proof of concept early study, we used the compassion engagement and action self-report scales (Gilbert et al., 2017) because they tap into the two aspects of compassion: “sensitivity” and “action”, in relationship to the flows of compassion: to self, to others, and from others. We were also interested in whether the energizing process impacts positive emotion in different ways. The “types of positive affect” scale enables the distinction among energizing positive emotion, relaxed, and also safeness-content positive emotion (Gilbert et al., 2009; Armstrong et al., 2021). One of the aspects of this type of compassion exercise was designed to help people experience being part of a compassionate community. To assess this aspect, we utilized the “social safeness and pleasure” scale which explores people's sense of being part of and secure within their social relational networks (Gilbert et al., 2009). Finally, we explored the impact on general wellbeing. Hence, in this early study, we were exploring the impact of energizing compassion on self-reported compassion, types of positive emotion, the degree to which it stimulated social connectedness and was associated with wellbeing. Subsequent studies will explore other potential effects.

### Aims

In this proof of principle research, we sought to explore if the research ideas of bringing energizing music to an adaptation of a tonglen practice are understandable and the methodology acceptable to participants. While objective-standardized matches can be used in such studies, what is especially important is qualitative research, which can also provide insight into the unique experiences, helpfulness or possible detrimental effect of the practices. In particular, we wanted to explore *how* people experienced energizing compassion that uses music and stimulates a sense of being part of a collective compassionate, motivated group. Hence, we incorporated a set of specifically designed single-item measures.

## Methods

### Design

The study employed a repeated measures within-subjects design using self-report measures and qualitative feedback before and after 2 weeks of practice.

### Participants

Initially, a study invitation was sent via email to the Compassionate Mind Foundation Google discussion list of ~1,600 members (mainly consisting of professionals interested in the evolutionary and biopsychosocial approach to compassion), inviting them to take part in the study. The only exclusion criterion was the inability to understand spoken and written English. Although many participants (*n* = 115) showed interest, only 43 participants completed measures both before and after using the practice for 2 weeks. The final group consisted of 35 female and 8 male participants aged 25–68 years (*M* = 49.35; *SD* = 11.06).

### Guided meditation

To some extent, the origins of this study were serendipitous. One of the authors (PG), a musician interested in the role of music to create emotional textures, had been practicing compassion exercises (such as the flow of life and tonglen practices), using different types of music. He identified one piece of music by Thomas Bergersen that, for him, generates energy for compassion. Out of curiosity, he offered to share his experience with participants at an online workshop to explore their experience. Participants were very enthusiastic and fed back that combining the music with this guided meditation generated feelings of being energized, connected, and joyful. With this anecdotal evidence, the authors decided to explore these experiences in a more standard scientific way.

The authors contacted Thomas Bergersen, composer of the music called *Final Frontier* from the album *Sun* (https://www.youtube.com/watch?v=BAzCf0ascW8), for permission to use the music with a guided meditation and it was granted for a small fee.

One of the authors (PG) then developed a video that provided a brief overview of the CFT evolutionary approach to compassion which was followed by guided meditation. This included the following information: 1. We, like all living things, have bodies, brains, and minds *that have been built for us, not by us*, to pursue survival and reproductive biopsychosocial goals (Gilbert, 1989). A lion did not choose to be a lion, and no zebra chose to be its prey. No human chose to be born human, nor did they choose their ethnicity, gender, birth, or cultural embeddedness. 2. Consequently, we inherit *tricky* brains that have the potential for love and compassion, but also hatred, callousness, and cruelty; we can act harmfully or helpfully. 3. Human history shows that we have a terrible dark side that has acted very harmfully through wars, slavery, exploitation, and oppression. 4. It is important to become mindfully aware of what our evolved and socially constructed brain can do through no fault of our own. 5. With awareness, comes the option to cultivate the most important motives that can help us stand against the motives behind the dark side of our mind (fear, rage, and greed)—this is the cultivation of compassion. Although given an evolutionary orientation (Gilbert, 1989, 2009, 2019a), this awareness of the challenges of the human mind has been articulated in Buddhist writings and others for many centuries (Dalai Lama, 1995; Austin, 2011). Hence, CFT focuses on addressing the dark side of the human mind.

Following this brief psychoeducation outline, participants were guided into the CFT grounding and body preparation for compassion meditation (see Gilbert and Simos, 2022). This involved attention to posture and brief soothing-rhythm breathing of around four breaths per minute. This led into the music and newly developed guided meditation based on tonglen practice, modified in the following ways:

- Rather than breathing in suffering and breathing out compassion, participants were guided to imagine breathing in a compassion-based white light or a mist that fills the body, then breathing out white light and mist, whilst imagining it reaching out into the world to address the suffering of others. Additionally, participants were guided to focus on feeling that this was something they really wanted to do, and how wonderful that would be if they could do it and help the world move toward a more compassionate orientation. This is linked to what is called a Bodhicitta wish (Rinpoche, 1999).
- The third component invited participants to imagine their social connectedness, to see themselves as part of a community of individuals working to address suffering in the world; to imagine all the white light they were sending out joining with others to fill the world with compassionate light. In Buddhist traditions, this can be thought of as connecting to a sense of being a member of a community sharing the same aspirations: a Sangha. Again, the focus is on the joyous and energizing experience of being part of such a community.

The full recording and transcript can be found at https://www.compassionatemind.co.uk/resource/audio.

### Procedure

Participants from the Compassionate Mind Foundation Google discussion group were recruited via email and directed to the study information sheet on Qualtrics (Qualtrics, Provo, UT). They provided written consent in accordance with the Declaration of Helsinki's ethical principles for medical research involving human subjects (World Medical Association, 2013).

Participants were then asked to complete two demographic questions regarding their age and gender, and two questions which explored their previous experiences of using compassion and mindfulness meditations. The latter two questions were rated on a 5-point Likert scale from 0 “not very much” to 4 “very much”. They were also asked to complete self-report questionnaires measuring compassion orientation, positive affect, wellbeing, and social safeness. Participants were subsequently emailed and given access to the video containing the overview of compassion and guided meditation, and invited to practice this over 2 weeks. They were invited to practice this as often as possible, with or without the recording and music.

After 2 weeks, the participants were invited via email to complete the same self-report measures completed initially, alongside some questions on usage and experience of the practice. These included several statements asking participants to rate the extent to which the meditation helped them feel more, for example, energized (measured from 1 “not at all” to 10 “very much”). Participants were also invited to complete a number of open-ended questions about their experience and how the meditation made them feel.

### Measures

Participants were asked to complete the following self-report measures both before and after 2 weeks of practice:

#### Three types of positive affect scale

Gilbert et al. (2008) developed this scale to measure the degree to which people experience different positive emotions. Participants are asked to rate 18 “feeling” words on a 5-point scale to indicate how characteristic it is of them (0 = “not characteristic of me” to 4 = “very characteristic of me”). Each item belongs to one of three subscales, which are Activated Positive Affect (e.g. “excited”), Relaxed Positive Affect (e.g. “peaceful”), and Safeness/Contentment Positive Affect (e.g. “secure”). The scale has good psychometric properties with Cronbach's alpha of 0.83 for Activating Positive Affect and Relaxed Positive Affect, and 0.73 for Safeness/Contentment Positive Affect (Gilbert et al., 2008).

#### Compassionate Engagement and Action Scales

The Compassionate Engagement and Action Scales (CEAS; Gilbert et al., 2017) are three scales that measure self-compassion (“I am motivated to engage and work with my distress when it arises”), the ability to be compassionate to distressed others (“I am motivated to engage and work with other peoples' distress when it arises”), and the ability to receive compassion (“Other people are actively motivated to engage and work with my distress when it arises”). In the first section of each scale, six items are formulated to reflect the six compassion attributes in the CFT model: sensitivity to suffering, sympathy, non-judgement, empathy, distress tolerance, and care for wellbeing. The second section of the scale has four more items that reflect specific compassionate actions to deal with distress. Participants are asked to rate each statement according to how frequently it occurs on a Likert scale from 1 to 10 (1 = “Never”; 10 = “Always”). The CEAS showed good to excellent internal consistencies of self-compassion engagement α = 0.74/action α = 0.89; for others engagement α = 0.81/action α = 0.88 and from others engagement α = 0.91/action α = 0.93 (Matos et al., 2021).

#### Social Safeness and Pleasure Scale

The Social Safeness and Pleasure Scale (SSPS; Gilbert et al., 2009) was developed to assess the extent to which individuals feel a sense of warmth, acceptance, and connectedness in their social world. Items include “I feel secure and wanted” and “I feel a sense of warmth in my relationships with people.” Participants rate their agreement with 11 statements using a Likert scale from 1 (“almost never”) to 5 (“almost all the time”). Previous research has found that this scale demonstrates good internal consistency (α = 0.96) (Kelly and Dupasquier, 2016).

#### Warwick and Edinburgh Well Being Scale

The Warwick and Edinburgh Well Being Scale (WEWBS; Tennant et al., 2007) is a 14-item scale assessing eudemonic and hedonic wellbeing. Items include cognitive processes (thinking clearly and solving problems), feelings (optimism, confidence, and feeling useful), and the quality of relationships with others (feeling loved and feeling close to other people). These are expressed as 14 statements which people can answer on a 5-point Likert scale (from 1 “none of the time” to 5 “all of the time”). Statements include “I've been feeling relaxed”, “I've been thinking clearly” and “I've been feeling loved”. The scale has good internal consistency (Cronbach's alpha score of 0.89 in a student sample and 0.91 in a sample representative of the population; Tennant et al., 2007).

#### Process

Following 2 weeks of practice, the participants were asked to reflect on the frequency of their usage of the meditation during the first and second week, with and without music, on a 5-point Likert scale (none, 1–2, 3–4, 5–6, or 7 or more times). The participants were asked to complete several single-item questions derived from common reflections people had made using the meditation. They were not derived in any specific order but simply developed to understand how the meditation was experienced. Participants were asked to rate the extent to which the practice made them feel more, for example, energized, joyful, and socially connected, on a 10-point Likert scale (from “not at all” to “very much”).

Participants were asked the following six open-ended questions exploring their experiences following 2 weeks of practice:

1. What were your standout experiences?
2. Can you describe how the practice made you feel?
3. Could you describe any impact the practice may have had on you?
4. Did you notice any change in your experience and understanding of compassion?
5. How do you think the practice might change the way you act in the future?
6. Any other feedback?

In addition, participants were invited to reflect on their experiences and share their observations from weeks 1 and 2. We analyzed this data separately from the six open-ended questions.

### Data analyses

#### Quantitative analysis

Data were analyzed using SPSS version 27. Item-level missing data were inputted using the mode for scales with fewer than 20% of items missing. In the case where missing data were higher, scale/item data were removed from the dataset; 62.6% of participants (*n* = 72/115) only completed the pre-measures. This left 37.4% (*n* = 43) of participants who completed all of the measures. This formed the basis of the analysis. Data were checked for normality and outliers; skewness and kurtosis values ranged within acceptable levels and no statistically significant violations were found (Kline, 2005). Means, standard deviations, and reliability statistics (Cronbach's alpha) were calculated for each study variable. Correlations were generated to explore relationships among the single-item measures. In addition, paired-samples *t*-tests explored the changes in questionnaire responses before and after the 2-week intervention. For the two questions pertaining to practice usage and engagement with the exercises, frequency analysis was conducted.

#### Qualitative analysis

Qualitative analysis of open-ended questions sought to explore the impact and experience of using energizing music and a guided variation of tonglen practice.

In consideration of the responses given in open-ended questions and the nature of this pilot study, qualitative content analysis (QCA) was used to explore the data. This provided an opportunity to explore theoretical issues, enhance understanding of the data (Elo and Kyngäs, 2008), provide inferences and insights from the data in this context, and highlight categories for further exploration (Krippendorff, 1980). The analysis, therefore, focused on the experience of energizing compassion as a new form of practice.

#### Qualitative content analysis process:

1. Preparation of the data and analysis of word frequency was carried out using MAXQDA 2022 (VERBI Software, 2021).
2. Inductive analysis of categories (open-ended question responses) was based initially on word frequency. Responses containing words with the highest frequency were then coded and grouped into categories. We then returned to all responses for each question to ensure themes had not been missed. The responses for each category were extracted and compiled into documents that covered each of the open-ended questions separately. This analysis sought to explore and identify critical processes (Lederman, 1991), with a qualitative focus on meanings, intentions, consequences, and context (Downe-Wamboldt, 1992).
3. Personal observations from weeks 1 and 2 were also analysed.
4. Analyses were reported using a combination of MAXQDA word cloud graphics (see Supplementary material) and categories highlighted in the inductive analysis. Some words were removed from the word cloud graphics to improve readability (e.g. “and”, “of”, “with” and “the”). A table of the process of analysis and the themes highlighted is also available in Supplementary material.

## Results

### Quantitative analysis

The majority of participants had some degree of experience with compassion (37/43 participants) and mindfulness practices (33/43 participants) as shown in Table 1.

**Table 1:** Participants' previous mindfulness and compassion meditation practice usage (*n* = 43).

	**0**	**1**	**2**	**3**	**4**
	**Not very much**				**Very much**
*To what extent do you use…* Compassion based practices?	2/43	4/43	10/43	15/43	12/43
Mindfulness and/or other meditations?	4/43	6/43	9/43	14/43	10/43

Participants were able to practice the meditation without music if they wished. The majority of participants reported that they practiced with the music three or more times in week 1 (30/41) and week 2 (23/41), (see Table 2).

**Table 2:** Participant engagement with the energizing compassion exercise with music during week 1 and 2 (*n* = 41; *n* = 2 missing).

	**None**	**1–2**	**3–4**	**5–6**	**7 or more**
Week 1	2/41	9/41	12/41	14/41	4/41
Week 2	7/41	11/41	8/41	12/41	3/41

Table 3 reveals that all the single item questions were highly correlated. Of interest, the experiences of feeling energised, joyful, socially connected and confident were highly correlated with the three flows of compassion. Interestingly too, courage had one of the highest correlation values, with wiseness and courageousness being very highly correlated. As single item measures, these are only indicative requiring more detailed analysis in the future.

**Table 3:** Descriptives (means and standard deviations) and correlations for single-item questions exploring the extent to which participants felt, for example energized, following two weeks' practice.

	**Mean**	**SD**	**1**	**2**	**3**	**4**	**5**	**6**	**7**	**8**	**9**	**10**
1. Energized	7.48	2.10	–									
2. Joyful	7.43	2.23	0.89^**^	–								
3. Socially connected	7.02	2.17	0.53^**^	0.56^**^	–							
4. Confident	7.12	2.20	0.79^**^	0.86^**^	0.68^**^	–						
5. Hopeful	7.43	2.20	0.86^**^	0.88^**^	0.64^**^	0.89^**^	–					
6. Compassionate to others	7.52	1.98	0.58^**^	0.58^**^	0.72^**^	0.67^**^	0.64^**^	–				
7. Compassionate to self	7.43	2.15	0.60^**^	0.57^**^	0.62^**^	0.65^**^	0.66^**^	0.92^**^	–			
8. Open to compassion from others	7.19	2.25	0.57^**^	0.60^**^	0.61^**^	0.74^**^	0.70^**^	0.79^**^	0.80^**^	–		
9. Courageous	7.10	2.36	0.81^**^	0.86^**^	0.53^**^	0.84^**^	0.89^**^	0.50^**^	0.56^**^	0.69^**^	–	
10. Wise	6.60	2.45	0.65^**^	0.72^**^	0.54^**^	0.81^**^	0.79^**^	0.40^**^	0.43^**^	0.66^**^	0.90^**^	–

** Correlation is significant at the 0.001 level (2-tailed).

(1) energized; (2) joyful; (3) socially connected; (4) confident; (5) hopeful; (6) compassionate to others; (7) compassionate to self; (8) open to compassion from others; (9) courageous; (10) wise.

Table 4 provides the data on paired samples *t*-tests (two-tailed) which were conducted to compare the pre- and post- questionnaire responses. After 2 weeks, there were significant increases in self-compassion, compassion to others, openness to compassion from others, activated positive affect, safe positive affect, social safeness, and wellbeing, with small to medium effect sizes. Differences in relaxed positive affect approached significance (*p* = 0.55).

**Table 4:** Descriptives (means and standard deviations) and paired sample *t*-test scores for questionnaire measure at pre- and post-2 weeks practice.

				***T*** **-test**			
	**α**	**Baseline**	**Post**	***t***	***df***	***p***	***d***
Compassion for self	0.86	70.44 (10.55)	79.44 (9.14)	−5.01	42	< 0.001	−0.76
Compassion to others	0.86	81.65 (8.75)	85.30 (7.02)	−3.18	42	< 0.005	−0.49
Compassion from others	0.96	66.77 (16.99)	72.42(15.06)	−2.41	42	< 0.05	−0.37
Social safeness	0.90	42.88 (7.02)	45.00 (7.29)	−1.99	42	≤ 0.05	−0.30
Activated positive affect	0.89	16.95 (6.44)	20.30 (6.24)	−4.14	42	< 0.001	−0.63
Relaxed positive affect	0.87	11.49 (4.63)	13.05 (4.75)	−1.97	42	0.055	
Safe positive affect	0.69	10.74 (2.60)	11.93 (2.63)	−2.82	42	< 0.01	−0.43
Wellbeing	0.92	50.30 (7.42)	54.14 (7.30)	−3.52	42	≤ 0.001	−0.54

Interestingly, those who practiced without the music three or more times had a change in self-compassion score of 6.64 in the first week whereas those who always practiced with the music had a change in score of 9.48.

### Qualitative content analysis

Results of the qualitative data analysis are reported here under the headings of each open-ended question with the following themes. All names are pseudonyms.

**1. What were your standout experiences?**

**Energy, energizing, exhilarating:** Participants reported an increase in energy and how this increase influenced their thinking after the experience.

*I noticed that on a couple of occasions I was surprised at the energy that I had which usually I wouldn't have. I even noticed that I had become more flexible and more aware of and not wanting to set into routines from which I would be reluctant to change. I realized I was more encouraging of myself to try different things*. Hetty

*Listening the first time - exhilarating, emotional, uplifting. Feeling energised after each practice and that I have more to offer than I give myself credit for*. Hilary

**Connection to self:** The experience encouraged participants to connect with themselves and find the motivation to “re-experience” positive and negative events. It also encouraged more appreciation of what they had to offer.

*This was a moving experience. I connect with my life and trying to re-experience the positive and negative events connected. I feel with more energy and I started planning workshops. I would like to learn more and to use these exercises with myself and others*. Veronika

**Connection to others:** Feeling a connection to others was reported in relation to “nameless/faceless” others, other participants in the study, colleagues, and “a sense of goodness in the world.”

*The emotional power of actually visualising others in the world doing this exercise and expending compassion to a nameless/faceless other. Because of the study, I felt a connection with the other participants of the study and was able to visualise others doing the same exercise as I and that helped me to accept compassion from others. There was one occasion where I had a difficult interaction with a colleague and utilised the exercise to extend compassion to them and the emotional connectedness and universal human experience I felt with them was very powerful to the point I became tearful. It helped me to see an alternative view of the disagreement and fix the situation*. Cleo

*A rousing sense of connectedness, a stirring of energy in my chest, feeling of being powerful with compassionate connection (rather than power in regards to others). A sense of goodness in the world*. Pat

**A global compassionate community:** Feeling connected to a global movement of compassionate others was described as “amazing,” “emotional”, and “beautiful.” Some participants reported feeling a transhistorical connection to “generations of human beings” with a sense of their contributions to “making life a little better for all of us”.

*Imagining being joined in a global circle of empowering and compassionate white light at various times throughout the day was an amazing experience*. Danielle

*I really enjoyed imagining being part of a whole movement of people all breathing out compassion into the world*. Lisa

*The meditation made me appreciate the effort and the contribution that humans have made, over the years. The music made me see generations of human beings, swarming like bees, all busy trying to make life a little better for all of us. And this is beautiful*. Kirsty

**2. Can you describe how the practice made you feel?**

**Part of a compassionate community:** The experience of feeling “united” and “belonging” was reported as powerful for many participants. Furthermore, participants emphasised how this made them feel more connected and hopeful.

*A sense of belonging, strong, and the unity of my whole light joining others'*. Kelly

*Like I am part of a compassionate world, I mostly felt that in my chest. I noticed my chest would actually expand and take up more space. It made me feel more hopeful for a compassionate world and positive about the world. It made me feel safer*. Pat

*Connected to compassionate motivation and part of a compassionate community*. Rita

*United, like a compassionate power ranger or another team of superheroes*. Sara

*It made me feel connected with others, both those doing the study and those others in the world who work daily to spread compassion. It made me feel more prepared for the rest of the day and able to take on anything that came my way*. Cleo

*Uplifted, joyful, hopeful, inspired by others, a strong sense of belonging*. Hilary

**Physical experiences:** Physical experiences as a result of, and during, the practice included “calm,” “warm,” “powerful,” “strengthened,” and “ALIVE.” One participant described how their experiences ranged from feeling “anxious and activated” to “calm and soothed.”

*Calm but energised, optimistic, ready*. Danielle

*It made me feel warm, as though my heart were expanding*. Grace

*Powerful, connected, courageous*. Ingrid

*It made me feel ALIVE and invigorated*. Lisa

*Sense of being physically and emotionally strengthened, grounded, nourished, determined*. Tina

*Energized. Connected with my body and centred. I enjoyed the music*... Veronika

*A range from anxious and activated to calm and soothed*. Wilma

*I felt that the music and energy can linger in the body especially when [I] think about it, certain rhythm and image comes up from the body, interconnected within self and the outside world*. Maria

**Changes in emotional experience over time:** Some participants reported feeling “overstimulated”, “overwhelmed” and “overloaded” with one participant describing a range of difficult emotions that they worked through (Carl). However, participants also noted over time that these challenges were reduced and became easier with practice.

*A whole range of emotions came up, fear, sadness, the feelings got better over the two weeks*. Carl

*A bit euphoric, on the edge of overstimulated. I have a pretty good acquired positive affect tolerance but I found it not quite but almost demanding to take in the intensity of the musical track*. Freire

*At first a little distressed and overwhelmed even when I adjusted the volume. After the first week I started enjoying the music*. Una

**3. Could you describe any impact the practice may have had on you?**

**Reminders and re-connection:** Perhaps unsurprisingly, given the global environment that participants found themselves in, many reported feelings of re-connection and being reminded that there are compassionate others in the world and they are not alone.

*I think that I've been struggling for the past 2 years with the pandemic, restrictions, isolation, war, callousness in the world, etc, and this practice helped me re-connect to a feeling that there are other things to be aware of – joy, collaboration, overcoming dark forces…we all have a dark side (or sides) but we can work together to make the world better*. Danielle

*A consolidated reminder that I am not alone!* Kelly

*I feel more connected to the world as a whole and the people within it. More hopeful*. Carl

*I liked the visualisation of white coming in and out as I was breathing, it made the practice more tangible for me. I live with the impact of trauma and it helped me to feel safer in that there are a lot of compassionate people in the world when I can be quite threat focused*. Pat

*I feel motivated to plan and practice this exercise. This exercise made me think about what is happening in other countries and the war and how other peoples are suffering right now. I feel the necessity of help and support and do something to make a better life*. Veronika

*It has lifted my energy levels and confidence to engage with others. I also have more space and energy to be compassionate to others again, not being debilitated by my own stress, anxiety and depression*. Elizabeth

*A lovely reminder that I am not alone - something to call upon and use to connect*. Hilary

**4. Did you notice any change in your experience and understanding of compassion?**

**Expanded understanding and appreciation for the dimensions of compassion:** Participants reported how the experience had expanded their understanding of, and appreciation for, experiencing a different dimension of compassion. Some participants noted the significance of experiencing compassion as a drive, and as activating, as opposed to previous experiences of soothing.

*Yes, compassion is being connected. It is also transcending the immediate reality*. Andrew

*A lot of the time I practice calming and soothing compassionate skills and this helped me to activate compassion focused drive rather than soothing. This is really very useful*. Pat

*Compassion is action... I want to do.. I would like to help*. Veronika

*I think the music directed me toward specific aspects of compassion that are not usually at the core of my awareness and practice; rather than empathy, connecting with suffering, loving kindness and being with the difficulty, trauma and suffering of humanity that is normally where my practice rests, I felt a much larger, expansive, joyful, fierce, transpersonal and cosmic level of compassion. I'm certain that reflects Gilbert's take on compassion and was refreshing and uplifting for me*. Anna

*It made me think that compassionate practice doesn't have to be slow. Excitement and enthusiasm within the music can still have compassionate qualities. I have used the white smoke visualisation while running for example. So the practice could contribute to exercise routines to calm anxiety and breathing*. Greta

**Embodied experience of compassion:** The embodied experience of compassion and its impact was highlighted by participants. Participants spoke of how the theoretical concepts of compassion became felt, and in turn, this aided in motivation and commitment to being compassionate.

*Yes, I have come to have a more energised, joyful, lighter sense of compassion - something I had been working on for a long time. The music paired with the CS practice helped me feel these things (as opposed to thinking about or wanting them)*. Danielle

*Grounding and a ‘felt’ or embodied sense of compassion*. Rita

*Yes. We are always telling our clients it's not their fault. For the first time, this did not just come across as a conceptual idea. I actually felt this for the first time this past week. I was able to be far more observant of my own process with an interested and curious attitude. I was able to provide myself with reassurance in a difficult client situation and....I ACTUALLY FELT REASSURED. I have had that happen before, but not like this. The reassurance felt....believable*. Eddie

*It helps to strengthen my understanding and embodiment of compassion, inhaling the sensitivity as awareness is also based on energy and exhaling to help the others which in return helps self too*. Maria

**5. How do you think the practice might change the way you act in the future?**

**Use exercise to develop own compassion practices:** Participants described how they hoped the exercise would help them to develop their own personal practices with others.

*Be more energetic with compassionate endeavours, not just calm and soothing*. Tina

*I would be well served to integrate these components into how I experience and practice compassion. it makes it much bigger than what I am able to generate and give, but tapping into a larger stream*. Anna

*Well, if things progress as they have, I suspect that doing this practice each day (I really look forward to it) can only strengthen my own sense of compassion, and my compassionate self, and motivate me to continue working to bring compassion to others. It has helped me slow down when I needed to address my own suffering. I think the biggest change is really feeling from the inside out reassured*. Eddie

*If the impact would remain each time, i believe with time would make me more calm, attentive, compassionate*. Jude

*Hopefully making me to do small compassionate things with more care and beauty*. Kirsty

*This is a practice of building a compassion mind and its neurological pathway. It will be reactivated whenever and where ever is needed*. Maria

**Call upon exercise for personal use:** The exercise was highlighted as something participants would return to when they were distressed.

*I think when I'm feeling stuck, afraid, beaten down, or just crappy, I can listen to it and re-connect to my inner compassionate warrior, or even imagine it lifting me*. Danielle

*I think it will protect me from feeling so low when distressed - less isolated*. Kelly

*I'll use these techniques to help myself cope with stressful situations and to moderate my responses to difficulty in future*. Elizabeth

*I think it will enhance my stamina, my sense of myself against challenges*. Hilary

**Use exercise to engage with others more:** Connecting with compassion being expressed by others was an area that participants thought the exercise would help them with.

*I would hope that it would help me grown in kindness and empathy toward others and myself. It would nice to release myself from the distress of being judgmental of myself and others and more loving*. Hetty

*Reminding me to draw on the compassion of others, even though I might not know, are putting compassion out into the world*. Ingrid

*Possibly connecting more easily, authentically and openly with others who are showing compassionate motivation and behaviours*. Rita

**6. Any other feedback?**

**Positive feedback:** Positive feedback reported changing views about compassionate motivation in participants themselves and others around the world.

*I am 100% glad that I had the opportunity to take part in this study. It has really changed how I feel about myself and the world around me. I am full of energy and enthusiasm which is a very welcome experience for me. I haven't felt like this for a number of years*. Olivia

**Helped with own and others' fears, blocks, and resistances (FBRs)**

*This practice has made me realize my struggle with myself where I couldn't imagine breathing out compassion towards others, because I couldn't accept that I could be a storehouse of compassion. And then I realized that even as I thought this about myself, I could feel compassion towards myself for feeling so badly about myself and that was a wonderful feeling!* Hetty

*Really helpful practice for strengthening determination and ability to keep bringing compassion into the world, especially when this is very challenging. The musical component in particular felt as if it helped me to replenish my energy and the sense of belonging to compassionate community strengthened my commitment and determination to keep going when compassion and connection feels very hard (is being unconsciously rejected/resisted)*. Tina

### Observations

The following section reports the participants' reflections and observations from weeks 1 and 2.

#### Observations from week 1

Participants engaged with the practice and communicated openly about their experiences. Some reported feeling energized by the music whilst others felt the music was too intense.

*The music felt inspirational. I imagined breathing compassion (in and out). I imagined others around the world being compassionate and imagined being connected to them. The music, however, didn't match my sense of compassion which is more tranquil*. Harry

*The music was music therapy - very powerful and evocative and made me imagine how your research team/Paul Gilbert imagines the properties of compassion - if I had to choose a piece of music to evoke compassion, it would not have chosen that one. very interesting to lean toward the dramatic, energizing, dynamic, expansive qualities within compassion as evoked in the music. my compassion mode is much more quiet, soft and tender, so it was an interesting stretch to enter the practice with the music. I appreciated what was evoked in me*. Freire

*I like the music better, the more I do it, and it is never the same meditation, there were always new images every time I do the meditation*. Kirsty

#### Observations from week 2

Participants continued to practice the exercise, with many reporting that they had engaged more with the music, were feeling more energetic, confident, and connected, and were adapting the exercise where they felt it was needed. There were also powerful reports of the influence of the practice on FBRs. Some participants described how they had used the practice over time, adjusted to the practice, or adjusted their thinking and understanding of the experiences they were having (see observation 2, below).

*I made more sense of the imagery this week! I think if I can't imagine it then it won't happen, that's the energising for me. I got an image of white light and could use it, that felt incredible. I enjoyed the music practise a lot more in the second week. I still wanted more of a choice of music (slower, medium or fast), the cut off crescendo was less distracting. I used the music on HiFi speakers without the verbal guiding, I'd memorised that, that was the most impactful experience*. Carl

*I noticed that doing the practice with the music was much more effective if I took additional time to practice beforehand; doing the 5 min practice (with music) alone was not the most powerful access to compassion, despite the music being evocative. it feels like 5 min of compassion practice of any kind is too short; more time allows me to really find a deeper connection with compassion and then the 5 min practice at that point is very accelerating*. Anna

*After the challenges of week 1 with accepting compassion from others I attempted to visualise this in conversations where I felt the other people expressing positive emotions to me. In these conversations I would visualise the stream of light and compassion coming from them to me. I found that this actually helped me to find the parts of the conversation and their behaviour that would indicate compassion and care that I would usually miss*. Cleo

*Yes, following up from the last box, I really have begun to feel far more compassionate toward myself as much as I am toward others. I have taken far more time to address my own suffering as it has arisen. Going into client sessions after having completed the practice, I have noticed being more engaged and open - far less tired. I really cannot overstate how much I have enjoyed and benefited from this practice of energizing compassion. To go from low motivation to address my own suffering to feeling that deep sense of belonging and connectedness and wanting to help myself...there is no better feeling*. Eddie

### Unique experiences—Connecting to suffering

It is important to keep in mind that compassion is about connecting to suffering and the first movement to suffering can be stressful and distressing (Di Bello et al., 2020). These were also themes and experiences that this practice stimulated for some participants. For some, the exercise seemed to connect to tuning into some of the global distress in the world linked to the war in Ukraine, the continuing COVID threat, and climate change, to name just a few. In addition, these practices can connect one to their own personal distress and, therefore, compassion practices that focus on bringing compassion to self and others need to be designed with an awareness of these effects, allowing participants to prepare beforehand.

*Really feeling energized was a stand-out point. I did the practice yesterday and there was a moment I just let myself cry. It wasn't because I was suffering, I think, it was because I think I felt so connected and grateful for just having that experience*. Eddie

*From day one the volume alteration that cut off in the third crescendo distracted me from the potential benefits. I found that I needed to do my usual 15-30 minute practiseso I could focus on compassion coming in before the practise with the music. Sobbed day 1,2 and 3. The imagery that connected to being at one with compassionate other developed*. Carl

*The meditation brought up two feelings for me I needed to somehow settle before engaging in the compassion meditation proper -firstly, grief about the state of the world, and second, a sense of distress about how little time/capacity I had to contribute beyond day to day work and parenting tasks. I had to use other compassion practices to help ground myself and develop some “wisdom” or perspective. One of the outcomes from this was to set myself a task each night to notice some activities in my day, however small, that made a compassionate contribution. The other thing was to expand on the visualisation and bring in more of a felt sense of tenderness/care. I was also aware that this practice differed from the traditional tonglen, where we engage more fully and experience in our own suffering and use this as a form of “exchange” with others*. Bethan.

## Discussion

This study explored a music-enhanced energizing compassion practice. It utilized an adapted form of tonglen. The focus was really three-fold: 1. To explore the impact of using energizing music; 2. The effects of the adapted form of tonglen for generating compassion motivation; and 3. The effect of imagining oneself as part of a compassion-focused community. Participants were invited to practice every day or most days with the music; the majority (around 70%) did so.

The self-report measures showed significant changes pre-to-post in the study variables. The effects included feeling energized, joyful, socially connected, hopeful, courageous, and wise (see Table 3). In addition, there were significant increases in self-compassion, compassion for others, compassion from others, activated positive affect, safe positive affect, social safeness, and wellbeing (see Table 4), with small to medium effect sizes. Differences in relaxed positive affect approached significance. Although the change scores for self-compassion without the use of music varied non-significantly, the degree of change is worthy of further investigation for future studies. Hence, this practice would seem to have a wide range of effects.

In regard to the experiential and qualitative findings, stand-out experiences included increased energy and connection to self and others. As noted in our report on the physical sensations, many experienced feelings of warmth, calmness, and strength. These themes arose in other parts of the interview too. For example, Hilary noted that listening for the first time felt “*exhilarating, emotional, uplifting. Feeling energised after each practice and that I have more to offer than I give myself credit for.”* There were also experiences of feeling strengthened and energized. For example, Danielle noted “*I think when I'm feeling stuck, afraid, beaten down, or just crappy, I can listen to it and re-connect to my inner compassionate warrior, or even imagine it lifting me.”* Participants reported that this “new” practice had enabled them to expand their understanding and appreciation for the dimensions of compassion. It gave them a more embodied experience of compassion. They also reported how they intended to continue using the practice in both a personal and professional capacity. Participants also noted an increased sense of social connectedness, belonging, and being part of a group of others, rather than pursuing compassion alone. The qualitative analysis indicated that many participants enjoyed using the music, however, nine participants did not like it. Consequently, we are exploring variations where individuals can choose their own music that will give them a sense of activation and enthusiasm in follow-up studies.

As often noted, the first movement to compassion is to address suffering. This can be distressing and stressful. Although we did not set out to explore this, some clients did note that they experienced distressing emotions when they connected to the realities of the human dark side and suffering in the world. Given the global environment that participants (along with all of us) experienced at the time of the study—war in Ukraine, the continuing COVID threat, and climate change—this distress is important to anticipate but equally not to be overwhelming.

## Limitations

This study recruited a small number of members from a compassion discussion list (*n* = 43) who were already familiar with the basic evolution-based compassion model. Indeed, only 4.7% of participants reported that they did not use compassion-based practices very much (for mindfulness and other meditation practices this was 9.3%), suggesting that the majority of participants in this group were regularly engaging in related practices. Subsequent research will therefore need to work with naïve participants and explore if the practice can have the same powerful effects. The small numbers also made it difficult to investigate specific effects like practicing with and without music. This will need to be addressed in subsequent studies. In addition, subsequent studies could invite clients to choose their own energizing music.

Another limitation inherent to studies incorporating self-report measures is the risk of demand characteristics biasing results. However, as this was a small proof-of-concept trial, it is hoped that the rich experiences reported as part of the qualitative analysis may help to support the quantitative responses. Subsequent studies may incorporate a single or double-blind design with a control group to mitigate against possible demand characteristics. There was little data collected from male participants. It is unclear whether this was because male participants showed less interest in the exercise than female participants, or whether this was a natural variation resulting from the sampling methods used. Future research should therefore aim to address this, so that we gain a better understanding of how male participants, in particular, experience the exercises.

## Conclusion

In summary, as a proof of concept, this study has shown the potential value of integrating energizing music with an energizing compassion focus, which had a positive impact on participants. Clearly, subsequent research will wish to identify and explore aspects of specific components. For example, to what degree did the energizing music or the sense of being part of a community impact results, and which aspects carried the most powerful impact? This study was not designed to explore that but rather whether this combination of energizing and engaging in a sense of community was acceptable to participants and of value. This is worthy of subsequent research including physiological and long-term effects.

## Data availability statement

The raw data supporting the conclusions of this article will be made available by the authors, without undue reservation. Available by contacting the corresponding author.

## Ethics statement

The studies involving human participants were reviewed and approved by University of Derby College of Health, Psychology and Social Care Research Ethics Committee. The participants provided their written informed consent to participate in this study.

## Author contributions

PG and JB were involved in all aspects of the study. PP, HG, and JB analyzed data. All authors contributed to the article and approved the submitted version.
