# Supplementary material for: Energizing compassion: using music and community focus to stimulate compassion drive and sense of connectedness
Source: Front Psychol. 2023 Oct 4;14:1150592. doi: 10.3389/fpsyg.2023.1150592 (PMC10586219; doi:10.3389/fpsyg.2023.1150592)

Energising Compassion. Supplementary Materials

**Figure 1.**

Standout experiences


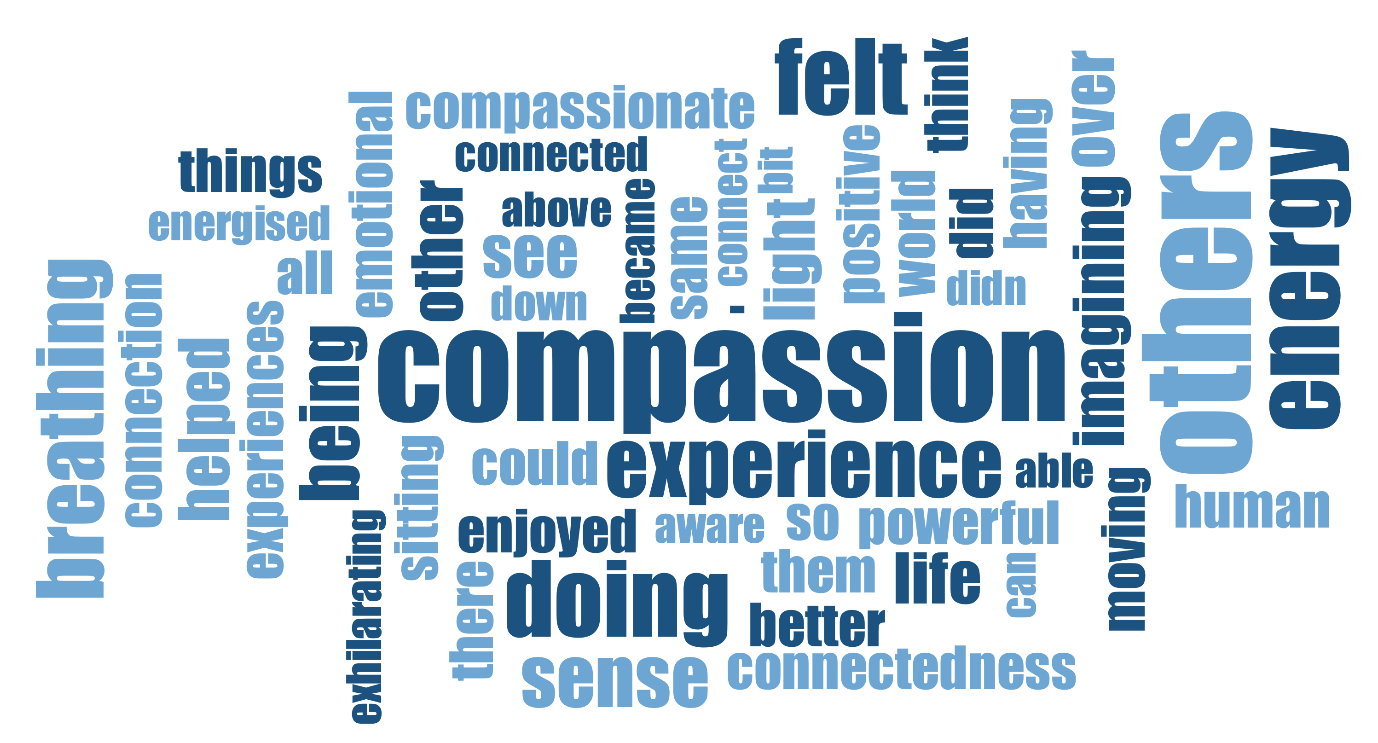


**Figure 2.**

Can you describe how the practice made you feel?


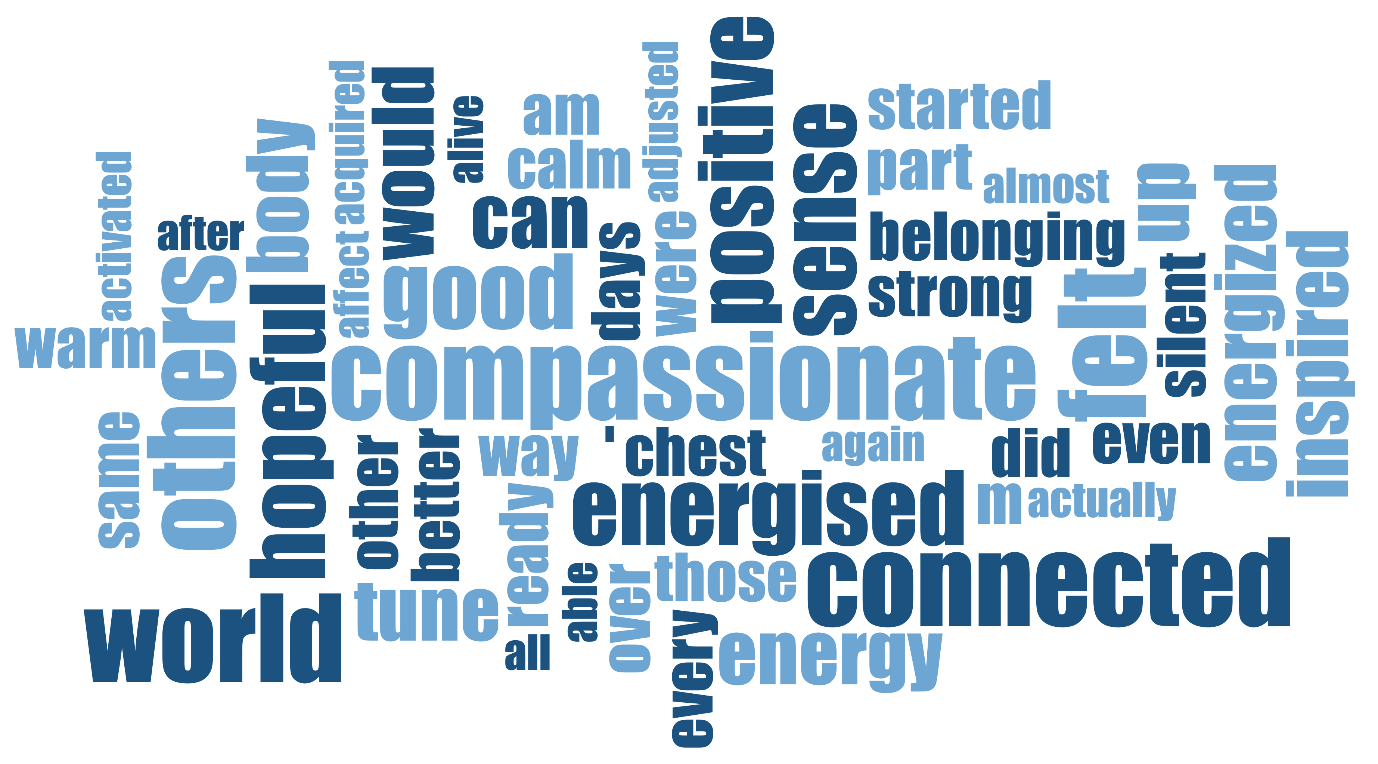


**Figure 3.**

Could you describe any impact the practice may have had to you?


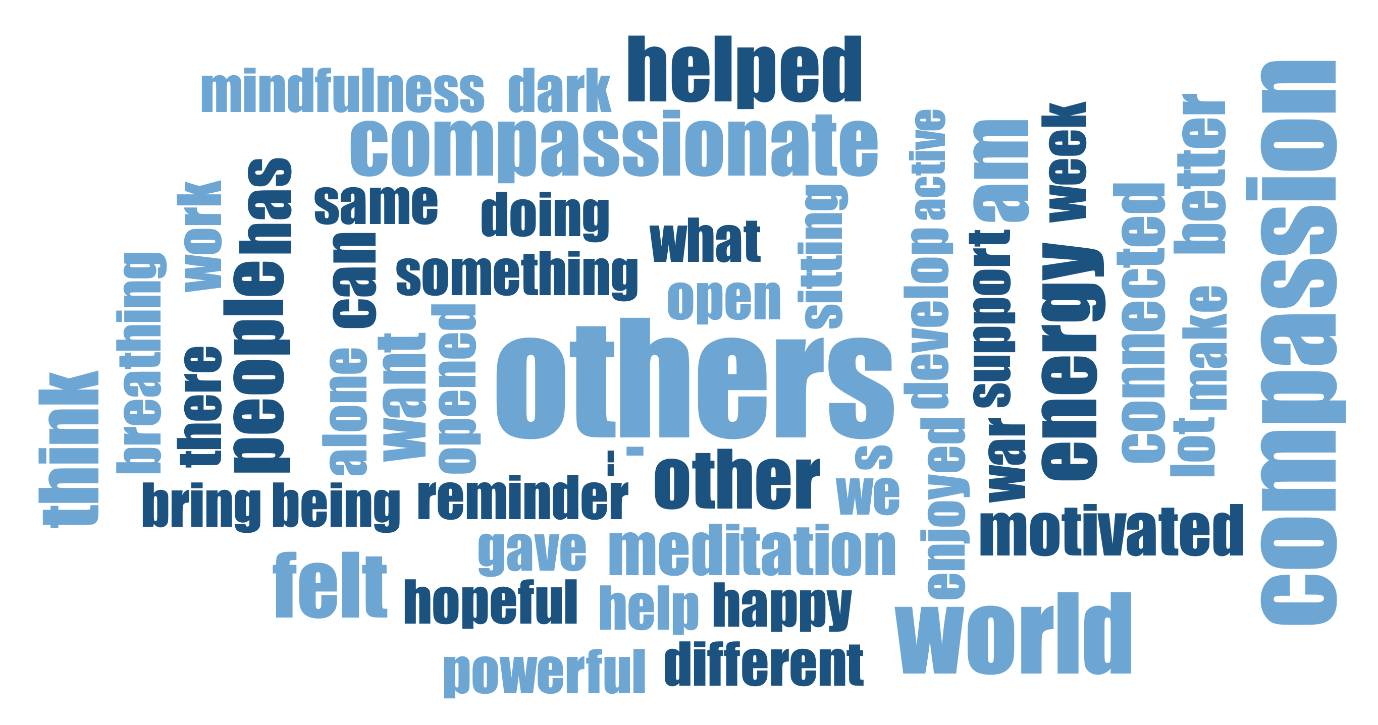


**Figure 4.**

Did you notice any change in your experience and understanding of compassion?


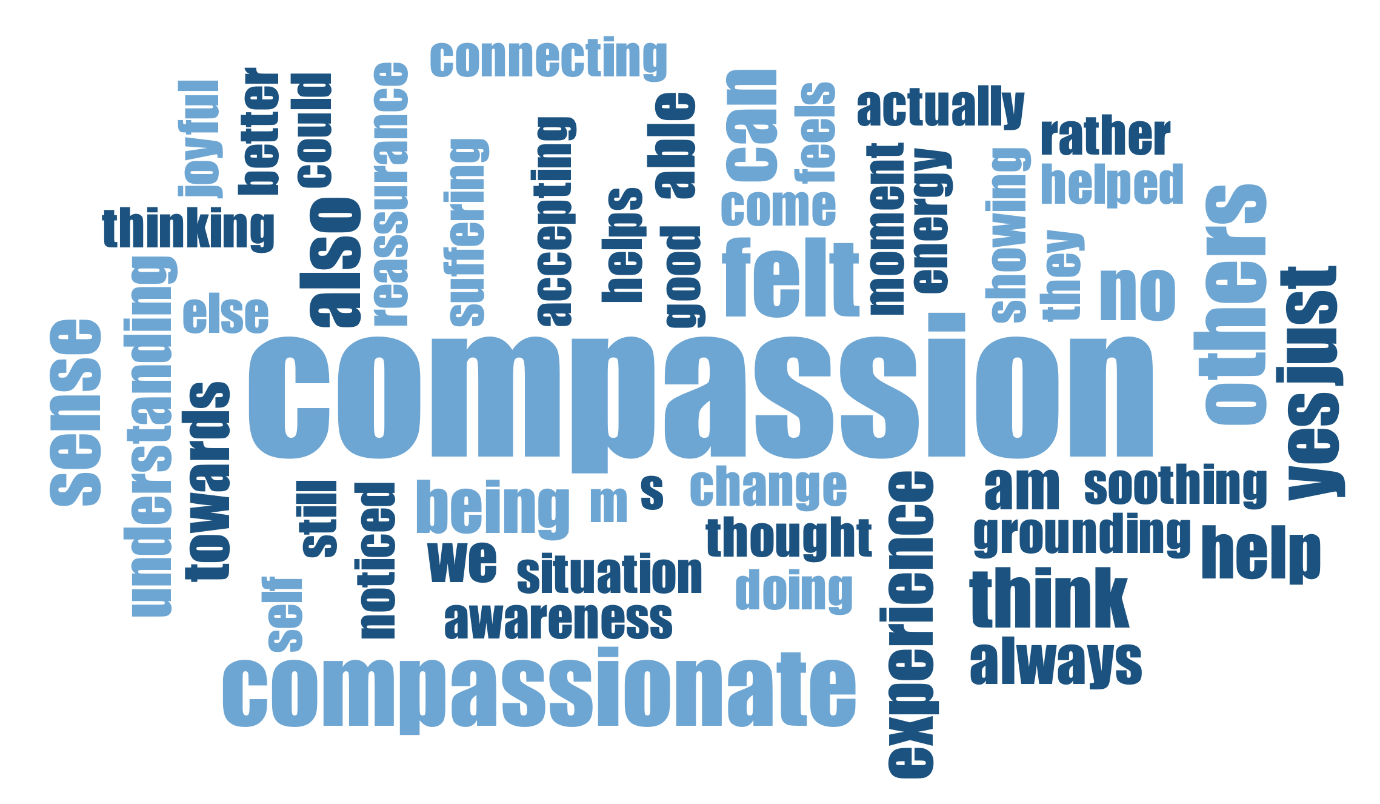


**Figure 5.**

How do you think the practice might change the way you act in the future?


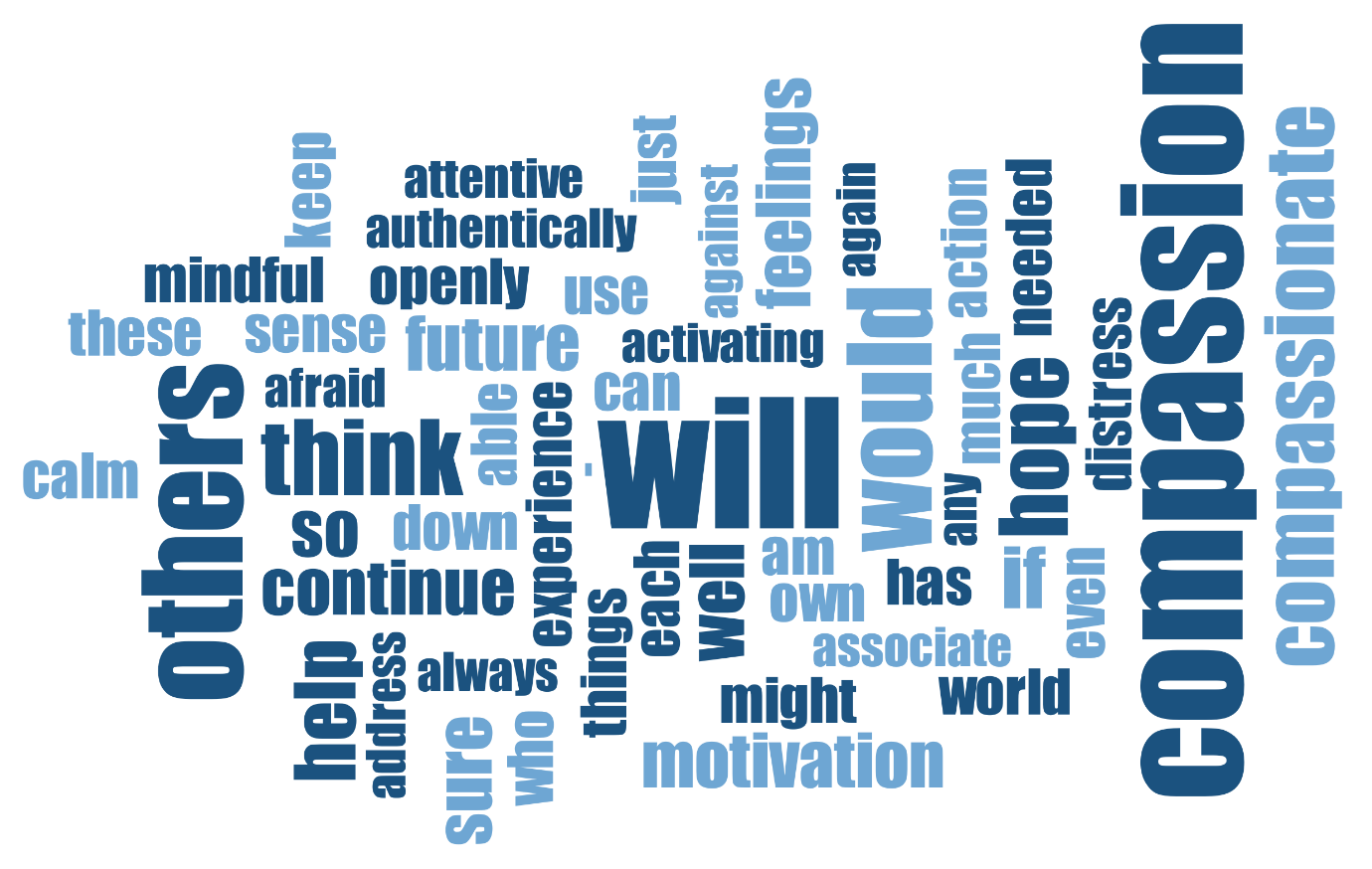

Supplement: Supplementary file 1 [file Data_Sheet_1.docx]
