# Supplementary material for: Energizing compassion: using music and community focus to stimulate compassion drive and sense of connectedness
Source: Front Psychol. 2023 Oct 4;14:1150592. doi: 10.3389/fpsyg.2023.1150592 (PMC10586219; doi:10.3389/fpsyg.2023.1150592)
Supplement: Supplementary file 2 [file Data_Sheet_2.docx]

**Supplementary materials.**

**Word frequency and Inductive analysis of open ended questions**

| **Open-ended Question** | **Word cloud: high frequency** | **Themes highlighted in Inductive analysis of responses** | **Example quotes** |
| --- | --- | --- | --- |
| What were your stand out experiences | Energy, energising, exhilarating | Increased energy | *I noticed that on a couple of occasions I was surprised at the energy that I had which usually I wouldn't have. I even noticed that I had become more flexible and more aware of and not wanting to set into routines from which I would be reluctant to change. I realized I was more encouraging of myself to try different things. Hetty* |
|  | Connection | Connection to self | *Listening the first time - exhilarating, emotional, uplifting. Feeling energised after each practice and that I have more to offer than I give myself credit for. Hilary* |
|  |  | Connection to others | *A rousing sense of connectedness, a stirring of energy in my chest, feeling of being powerful with compassionate connection (rather than power in regards to others). A sense of goodness in the world. Pat* |
|  | Compassion | A global compassionate community | *Imagining being joined in a global circle of empowering and compassionate white light at various times throughout the day was an amazing experience. Danielle* |
| Can you describe how the practice made you feel? | United, belonging. | Part of a compassionate community | *It made me feel connected with others, both those doing the study and those others in the world who work daily to spread compassion. It made me feel more prepared for the rest of the day and able to take on anything that came my way. Cleo* |
|  | Calm, warm, powerful, strengthened, ALIVE. | Physical experiences | *Sense of being physically and emotionally strengthened, grounded, nourished, determined. Tina* |
|  | Overstimulated, overwhelmed, overloaded. | Difficult emotional experiences | *A whole range of emotions came up, fear, sadness, the feelings got better over the two weeks. Carl* |
| Could you describe any impact the practice may have had on you? | Connection, people, not alone | Reminders and re-connection | *I think that I've been struggling for the past 2 years with the pandemic, restrictions, isolation, war, callousness in the world, etc, and this practice helped me re-connect to a feeling that there are other things to be aware of -- joy, collaboration, overcoming dark forces. The part about joining my light with others reminded me of the end of Buffy the Vampire Slayer, and you may laugh but I always felt the story of Buffy and her comrades was deeply rooted in archetypes and hopeful about the fact that we all have a dark side (or sides) but we can work together to make the world better. Danielle* |
| Did you notice any change in your experience and understanding of compassion? | Connection, drive, action, uplifting | Expanded understanding and appreciation for the dimensions of compassion | *A deepening of it to another dimension that can be freeing, light-hearted, not just very grounding and gentle and now knowing the somatic experience of that to use moment to moment. Hilary* |
|  | Feel, felt, embodied | Embodied experience of compassion | *Grounding and a ‘felt’ or embodied sense of compassion. Rita* |
| How do you think the practice might change the way you act in the future? | Energetic, strengthen, building | Use exercise to develop own compassion practices | *I would be well served to integrate these components into how I experience and practice compassion. it makes it much bigger than what I am able to generate and give, but tapping into a larger stream. Anna* |
|  | Stuck, low, difficulty | Call upon exercise for personal use | *I'll use these techniques to help myself cope with stressful situations and to moderate my responses to difficulty in future. Elizabeth* |
|  | Others | Use exercise to engage with others more | *Reminding me to draw on the compassion of others, even though I might not know, are putting compassion out into the world. Ingrid* |
